# Clathrin-Mediated Endocytosis Delivers Proteolytically Active Phytaspases Into Plant Cells

**DOI:** 10.3389/fpls.2019.00873

**Published:** 2019-07-18

**Authors:** Svetlana V. Trusova, Anastasia D. Teplova, Sergei A. Golyshev, Raisa A. Galiullina, Ekaterina A. Morozova, Nina V. Chichkova, Andrey B. Vartapetian

**Affiliations:** ^1^Department of Chemistry and Biochemistry of Nucleoproteins, Belozersky Institute of Physico-Chemical Biology, Moscow State University, Moscow, Russia; ^2^Faculty of Bioengineering and Bioinformatics, Moscow State University, Moscow, Russia

**Keywords:** plant cell death, clathrin-mediated endocytosis, protein localization, proteolytic activity, subtilisin-like protease, phytaspase

## Abstract

Phytaspases belong to the family of plant subtilisin-like proteases and are distinct from other family members, as they have strict and rarely occurring aspartate cleavage specificity and unusual localization dynamics. After being secreted into the apoplast of healthy plant tissues, phytaspases are able to return back into cells that have been committed to cell death due to a variety of biotic and abiotic stresses. It was recently discovered that retrograde transport of phytaspases involves clathrin-mediated endocytosis. Here, consequences of phytaspase internalization were studied. Proteolytic activity of phytaspases in the apoplast and intracellular protein fractions obtained from *Nicotiana benthamiana* leaves containing either endogenous phytaspase only or transiently producing *Nicotiana tabacum* phytaspase-EGFP protein (*Nt*Phyt-EGFP) was determined. We demonstrated that triggering phytaspase internalization by antimycin A-induced oxidative stress is accompanied by re-distribution of phytaspase activity from the apoplast to the cell interior. Inhibition of clathrin-mediated endocytosis by co-production of the Hub protein prevented phytaspase internalization and phytaspase activity re-localization. Specificity of endocytic uptake of phytaspases was demonstrated by the co-production of an apoplast-targeted mRFP protein marker, which retained its apoplastic localization when phytaspase internalization was essentially complete. Overproduction of *Nt*Phyt-EGFP, but not of the proteolytically inactive phytaspase mutant, *per se* caused moderate damage in young *Nicotiana benthamiana* seedlings, whereas antimycin A treatment induced a pronounced loss of cell viability independent of the *Nt*Phyt-EGFP overproduction. Interestingly, inhibition of clathrin-mediated endocytosis abrogated cell death symptoms in both cases. In contrast to stress-induced internalization of tobacco phytaspase, *Arabidopsis thaliana* phytaspase-EGFP protein (*At*Phyt-EGFP) was spontaneously internalized when transiently produced in *N. benthamiana* leaves. The *At*Phyt-EGFP uptake was dependent on clathrin-mediated endocytosis as well, the internalized protein being initially visualized within the membranous vesicles. At later time points, the EGFP tag was cleaved off from *At*Phyt, though the elevated level of intracellular *At*Phyt proteolytic activity persisted. Our data, therefore, point to clathrin-mediated endocytosis as a means to deliver proteolytically active phytaspases into plant cells. It would be interesting to learn whether or not phytaspases are unique among the large family of plant subtilisin-like proteases in their ability to utilize retrograde trafficking.

## Introduction

Phytaspases belong to the vast family of plant subtilisin-like proteases (subtilases), which includes many members in each plant species, e.g., 56 in *Arabidopsis thaliana* (Rautengarten et al., 2005), 63 in rice (*Oryza sativa*, Tripathi and Sowdhamini, 2006), and 82 in grape (*Vitis vinifera*, Cao et al., 2014; Figueiredo et al., 2016) and tomato (*Solanum lycopersicum*, Reichardt et al., 2018). Subtilases are known to be involved in diverse processes, from unselective protein degradation (Yamagata et al., 1994; Hamilton et al., 2003) to precise processing of precursor proteins (Liu et al., 2007; Liu and Howell, 2010; Sénéchal et al., 2014; Ghorbani et al., 2016; Schardon et al., 2016; Beloshistov et al., 2018). However, function of the majority of these proteolytic enzymes remains unknown. Similar to other plant subtilases, phytaspases are synthesized as proteolytically inactive precursor proteins, which possess an N-terminal signal peptide, a prodomain, and a peptidase domain (Chichkova et al., 2010; Schaller et al., 2018). The precursor protein is autocatalytically and constitutively processed, and the mature proteolytically active enzyme is released into the apoplast (Chichkova et al., 2010), which is also typical for plant subtilases. However, phytaspases differ from other subtilases in two ways. First, phytaspases display strict aspartate (Asp) specificity of hydrolysis. The efficiency of hydrolysis after Asp residue strongly depends on the preceding three amino acid-long motif, which confers strong selectivity of phytaspase-mediated protein fragmentation. For native rice phytaspase, the preferred upstream recognition motif is remarkably hydrophobic (Galiullina et al., 2015). Computer-based modeling of the *Nicotiana tabacum* phytaspase with its peptide inhibitor provided an explanation for Asp specificity of phytaspases (Vartapetian et al., 2011). A direct consequence of Asp specificity is observed at the prodomain-peptidase domain junction in the phytaspase precursor protein. The C-terminal residue of prodomain is Asp, which is consistent with the self-processing mode of generation of the mature enzyme. Mutating this junction Asp residue precludes processing/activation of the phytaspase precursor and the release of the mature enzyme into the apoplast (Chichkova et al., 2010). The presence of junction Asp residue may serve as a phytaspase signature within the plant subtilase family, and this sign has been successfully used to identify phytaspase-encoding genes in several plant species. The number of phytaspase genes appears to vary in plant genomes, from a single gene in *A. thaliana* to 12 in *S. lycopersicum* (Chichkova et al., 2018; Reichardt et al., 2018).

The second distinctive characteristic of phytaspases is their dynamic localization. The apoplast is not the end point of phytaspase trafficking. Phytaspases are known to be crucial to the implementation of programmed cell death (PCD) in plants triggered by biotic and abiotic stresses. Increased phytaspase levels were shown to enhance stress-induced and spontaneous plant cell death, whereas down-regulation of phytaspase activity suppressed cell death (Chichkova et al., 2004, 2010; Reichardt et al., 2018). Upon the induction of cell death, phytaspases become physically re-localized from the apoplast toward the cell interior (Chichkova et al., 2010, 2012). This retrograde transport is considered to be unique among plant subtilisin-like proteases. The involvement of clathrin-mediated endocytosis in phytaspase internalization has recently been documented (Trusova et al., 2019), which provokes important questions regarding possible mechanisms and consequences of retrograde phytaspase trafficking.

Here, we examined possible correlations between clathrin-mediated phytaspase re-entry into plant cells and proteolytic activity of phytaspases both outside and inside plant cells. We found that clathrin-mediated endocytosis provides a gateway for delivery of proteolytically active phytaspases into plant cells. Also, results from this study suggest that a specific recognition mechanism for phytaspase internalization may exist. Finally, our study points to the importance of clathrin-mediated endocytosis for the accomplishment of antimycin A-induced and phytaspase overexpression-promoted plant cell death.

## Materials and Methods

### Plant Growth Conditions

*Nicotiana benthamiana* plants were grown at 25°C in soil in a controlled environment under a 16 h/8 h day/night cycle. Protein transient expression was performed using 6-week-old plants. For evaluation of cell death symptoms, *Nicotiana benthamiana* seedlings were grown on the half-strength Murashige and Skoog medium (pH 5.7) containing 1% glucose and 0.8% agar.

### Plasmid Construction

For construction of recombinant *Nt*Phyt-EGFP, *Nt*Phyt-S537A-EGFP, *Nt*Phyt-mRFP, *At*Phyt-EGFP, and *At*Phyt-S553A-EGFP fusion proteins, the downstream GST tag gene in the previously described Phyt-GST constructs within the pLH7000 binary vector backbone (Chichkova et al., 2010, 2018) was substituted with the PCR-amplified EGFP or mRFP gene. To create the SP-mRFP fusion protein, the signal peptide-encoding region of the *Nt*Phyt cDNA was PCR-amplified and ligated upstream of and in frame with the mRFP gene between the *Nco*I and *Sac*I sites of the pLEX7000 expression vector (Beloshistov et al., 2018). The mRFP gene alone was inserted in the same vector in an analogous fashion to serve as a control. To obtain the mRFP-Hub1 fusion protein, a pCambia1300-derived expression vector pCambia1300EX was constructed by inserting the 1,200 bp long *Sal*I-*Eco*RI DNA fragment of pLEX7000 encompassing the dual 35S promoter, a polylinker and a transcription terminator between the *Sal*I and *Eco*RI sites of the pCambia1300 binary vector. cDNA encoding the C-terminal fragment of *A. thaliana* clathrin heavy chain 1was PCR-amplified using primers 5′-CCAGGATCCAAGAAGTTTAACTTAAATGTTCAG-3′ and 5′-GTTGGTACCTTAGTAGCCGCCCATCGGTG-3′. Hub1 cDNA (*c*. 1,870 bp long) was then inserted downstream of and in frame with the mRFP gene between the *Nco*I and *Kpn*I sites of the pCambia1300EX binary vector. The EGFP-LTI6b-encoding plasmid was a gift from M. Taliansky (The James Hutton Institute, UK).

### Agroinfiltration and Protein Fractionation

**Figure 1:**
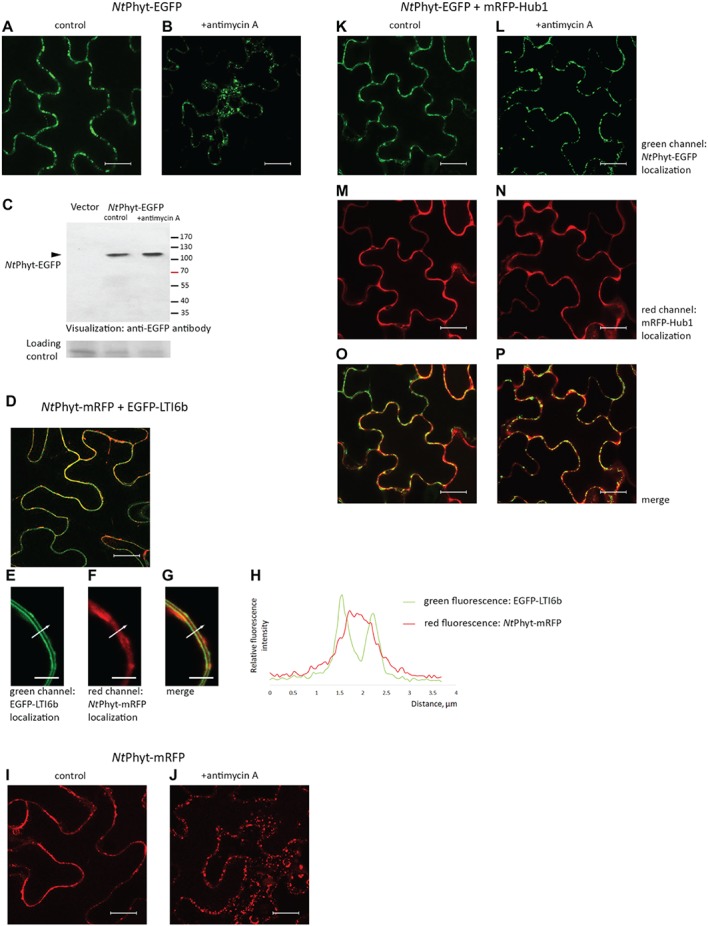
Re-localization of the *Nt*Phyt-EGFP protein in *N. benthamiana* leaves upon induction of oxidative stress depends on clathrin-mediated endocytosis. Confocal microscopy visualization of the *Nt*Phyt-EGFP protein in non-stressed leaves **(A)** and in antimycin A-treated leaves (10 μM antimycin A for 14 h, **B**). **(C)** The *Nt*Phyt-EGFP protein (~110 kDa) is not degraded in response to antimycin A treatment. Western blot analysis of protein extracts obtained from mock-treated (control) and antimycin-treated leaves (+antimycin A) using anti-EGFP antibody. “Vector,” the empty vector control from leaves without *Nt*Phyt-EGFP production. The lower panel, the loading control depicting the Coomassie blue-stained Rubisco band. **(D)** Co-production of *Nt*Phyt-mRFP and EGFP-LTI6b visualizes both proteins at the cell borders. In **(D–G)**, the agro-infiltrated tissues were plasmolysed with 0.5 M mannitol, 15 min before confocal analysis, to demonstrate largely apoplastic fluorescence of *Nt*Phyt-mRFP versus the plasma membrane-localized fluorescence of EGFP-LTI6b. Bar, 5 μm for **(E–G)**. **(H)** Measuring green (EGFP-LTI6b) and red (*Nt*Phyt-mRFP) fluorescence intensities across the boundaries of the adjacent cells (shown by the arrow in **E–G**), which shows that red fluorescence is peaking between the plasma membranes. **(I,J)** The *Nt*Phyt-mRFP protein is localized similarly to *Nt*Phyt-EGFP both before **(I)** and after **(J)** overnight treatment with 10 μM antimycin A. **(K–P)** Co-production of *Nt*Phyt-EGFP together with mRFP-Hub1 prevents *Nt*Phyt-EGFP internalization in response to antimycin A treatment. Of note, no discernible signal was detected in **(A)** and **(B)** in the red channel with the current settings in the absence of mRFP production. Bar, 20 μm for images **(A,B,D,I–P)**.

**Figure 2:**
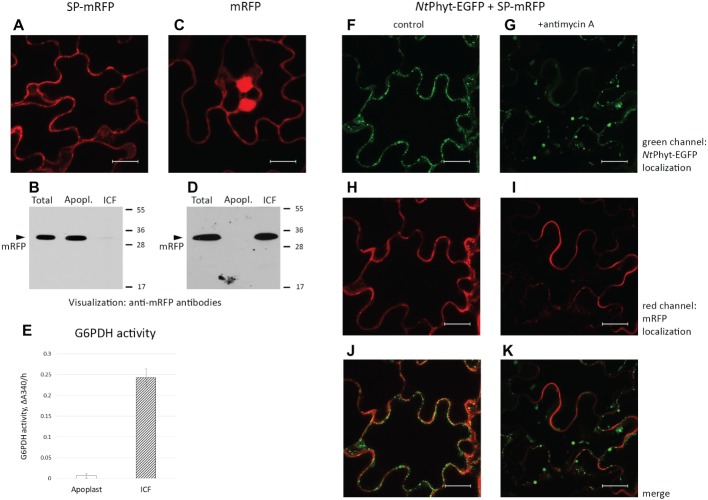
Signal peptide-mRFP (SP-mRFP) as a soluble apoplastic protein marker. **(A)** Confocal fluorescence microscopy of *N. benthamiana* leaves producing SP-mRFP visualizes mRFP fluorescence at the cell borders. **(B)** Western blot analysis of mRFP (~30 kDa) distribution between the apoplastic (“Apopl.”) and intracellular (“ICF”) protein fractions. “Total” represents the leaf extract without fractionation. Anti-mRFP antibodies were used for protein detection. Positions of molecular weight protein markers are indicated on the right. **(C)** Fluorescence microscopy localization of free mRFP synthesized in *N. benthamiana* leaves. Bar in **(A)** and **(C)**, 20 μm. **(D)** Western blot analysis demonstrating intracellular localization of free mRFP. Designations as in **(B)**. **(E)** Determination of glucose 6-phosphate dehydrogenase (G6PDH) activity in the apoplastic (“Apoplast”) and intracellular (“ICF”) protein fractions. The activity was determined spectrophotometrically, by measuring change in absorbance at 340 nm/h (ΔΑ340/h). Average ± SD for three independent experiments with two replicates in each. **(F–K)** Using SP-mRFP as a marker to assess specificity of *Nt*Phyt-EGFP internalization. Confocal fluorescence microscopy of *N. benthamiana* leaves co-producing *Nt*Phyt-EGFP and SP-mRFP. The left column shows non-stressed leaves and the right column shows leaves treated with 10 μM antimycin A for 14 h. Bar, 20 μm.

**Figure 3:**
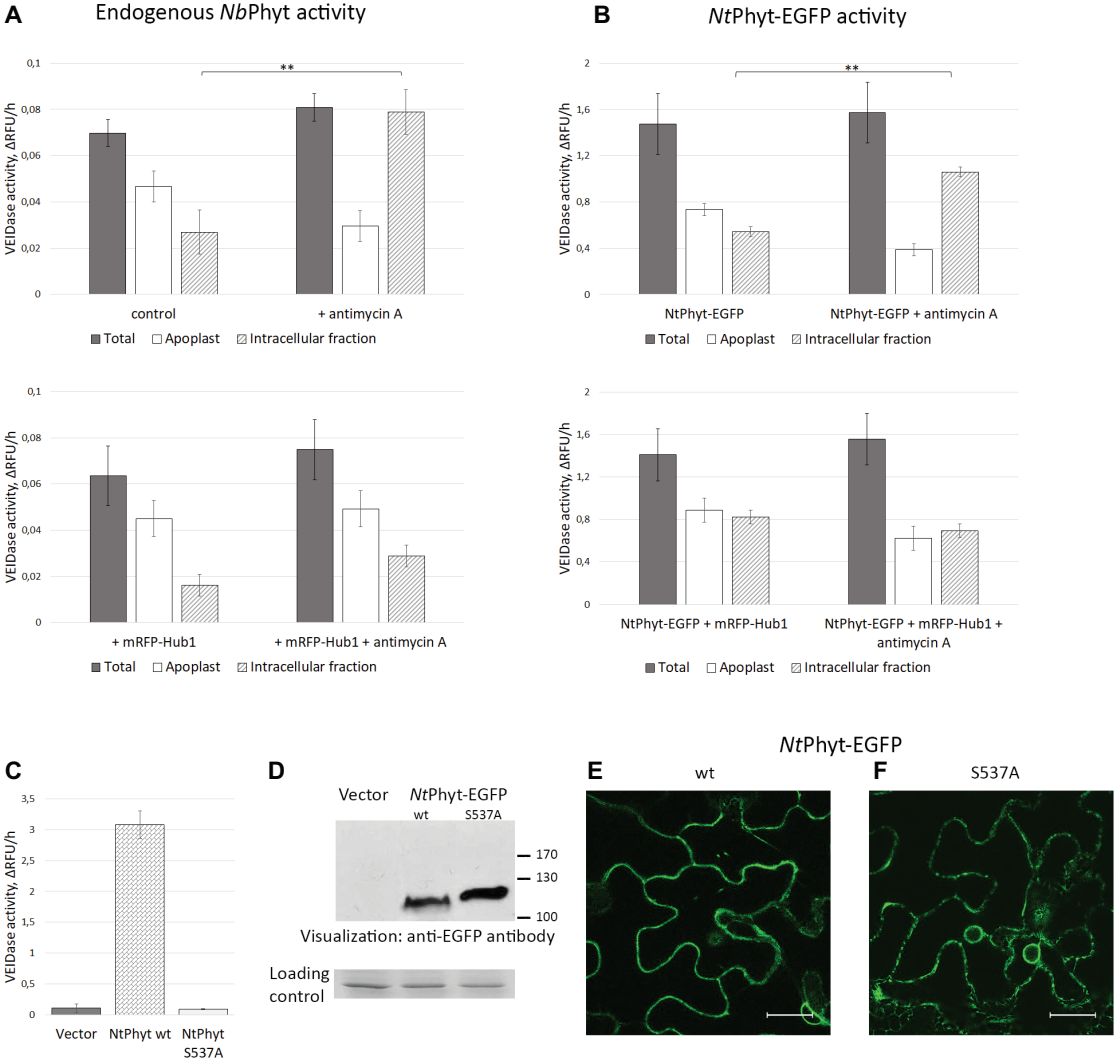
The internalized *Nt*Phyt-EGFP and endogenous *N. benthamiana* phytaspases are proteolytically active. Endogenous *Nb*Phyt **(A)** and ectopic (*Nt*Phyt-EGFP, **B**) phytaspase activity in the extracellular (“Apoplast”) and intracellular fractions obtained from non-stressed leaves and from antimycin A-treated leaves that either produce mRFP-Hub1 (lower panels) or do not produce mRFP-Hub1 (upper panels). “Total” represents the leaf extract without fractionation. +antimycin A, samples from antimycin A-treated leaves (10 μM antimycin A for 14 h). Control, samples from water-infiltrated leaves. Phytaspase activity was analyzed using 30 μM Ac-VEID-AFC fluorogenic peptide as a substrate. Relative rates of hydrolysis were determined as an increase of relative fluorescence units per hour (ΔRFU/h). Data represent the mean of three experiments ±SD. Significant differences are shown as **p < 0.01 (Student’s *t*-test). Samples in **(A)** and **(B)** were prepared in an identical fashion. Note that ‘total’ activity values in **(B)** are approximately 20-fold higher than in **(A)** due to *Nt*Phyt-EGFP production. **(C)**, production of *Nt*Phyt-EGFP in *N. benthamiana* leaves results in a significant increase of the Ac-VEID-AFC-hydrolyzing activity observed in total protein extracts, whereas production of the catalytically inactive phytaspase mutant (*Nt*PhytS537A-EGFP) does not. “Vector” sample from leaves infiltrated with agrobacteria carrying empty vector. Proteolytic activity was determined as in **(A)** and **(B)**. Data represent the mean of three independent experiments ±SD. **(D)** Western blot analysis of extracts used in **(C)** with anti-EGFP antibody to confirm comparable levels of production and accumulation of the *Nt*Phyt-EGFP (wt, ~ 110 kDa) and *Nt*Phyt-S537A-EGFP (S537A, ~ 120 kDa) proteins. The lower panel, the loading control depicting the Coomassie blue-stained Rubisco band. **(E,F)** Confocal microscopy images of *N. benthamiana* cells producing *Nt*Phyt-EGFP (wt) and *Nt*PhytS537A-EGFP (S537A). Bar, 20 μm.

**Figure 4:**
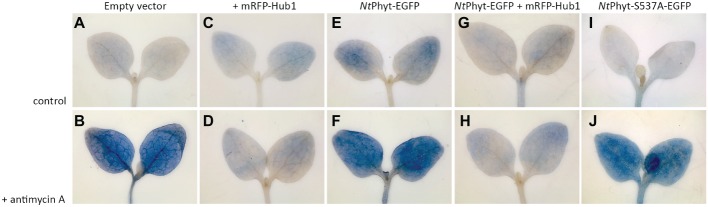
Inhibition of clathrin-mediated endocytosis in *N. benthamiana* seedlings by mRFP-Hub1 production opposes cell death. Seven-day-old seedlings were agroinfiltrated to produce mRFP-Hub1 **(C,D)**, *Nt*Phyt-EGFP **(E,F)**, both proteins together **(G,H)**, or neither of them (empty vector, **A** and **B**), and *Nt*Phyt-S537A-EGFP **(I,J)**. Three days after agroinfiltration, the seedlings were treated with 10 μM antimycin A for 3 h (the lower row) or mock-treated (buffer only control, the upper row). Subsequent staining with Evans Blue revealed marked cell death in the cotyledons upon antimycin A treatment **(B,F,J)**, which was prevented by the mRFP-Hub1 production **(D,H)**. Overproduction of *Nt*Phyt-EGFP caused moderate damage by itself **(E)** and again, co-expression of mRFP-Hub1 nullified this effect **(G)**. Overproduction of proteolytically inactive *Nt*Phyt-S537A-EGFP mutant failed to cause cell death in the absence of antimycin A treatment **(I)**. Data were reproducible over three independent experiments.

**Figure 5:**
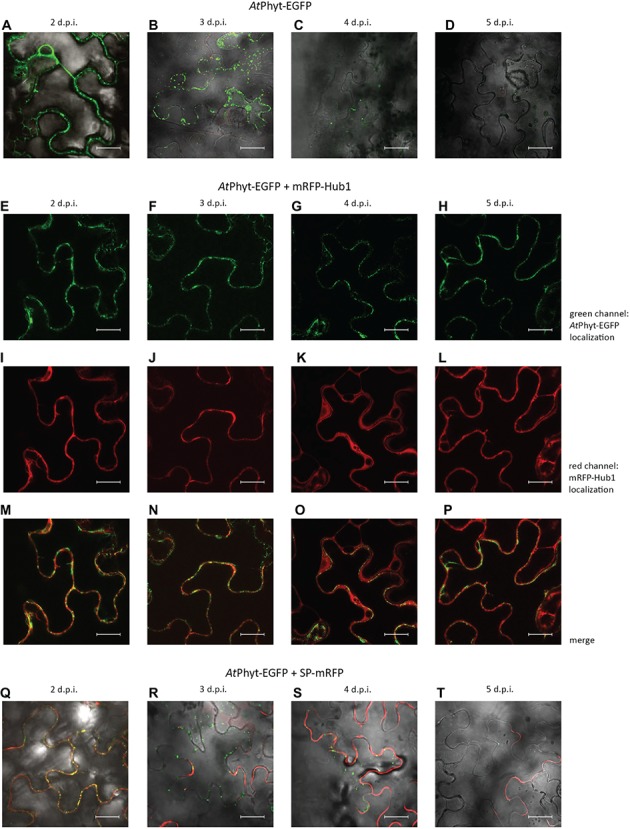
Localization dynamics of *At*Phyt-EGFP in *N. benthamiana* leaves: confocal microscopy examination. The upper row **(A–D)** shows leaves producing only *At*Phyt-EGFP at different days post-infiltration (d.p.i., indicated at the top). Internalization of *At*Phyt-EGFP is most clearly visible at day 3 p.i. **(B)**, after which the signal intensity drops dramatically. **(E–P)** show leaves co-producing *At*Phyt-EGFP and mRFP-Hub1 at different d.p.i. Note that no spontaneous internalization of *At*Phyt-EGFP occurred in the presence of Hub1 at any time point. **(Q–T)** show leaves co-producing *At*Phyt-EGFP and SP-mRFP at different d.p.i., demonstrating specificity of *At*Phyt-EGFP internalization. For **(A–D)** and **(Q–T)**, signals in red channel, green channel, and bright field were merged. Bar, 20 μm.

**Figure 6:**
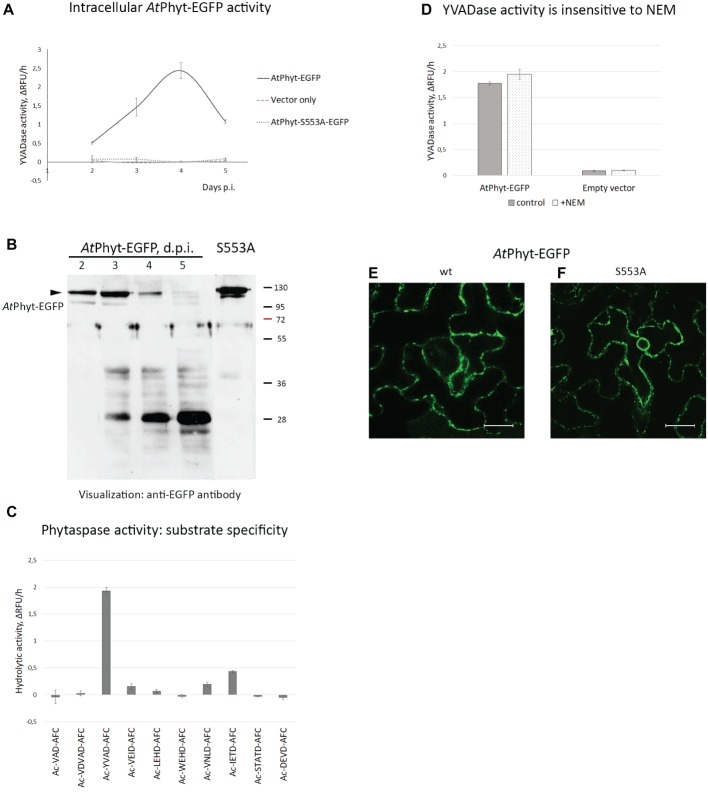
Origin of phytaspase activity in *At*Phyt-EGFP producing *N. benthamiana* leaves. **(A)** Determination of phytaspase activity in intracellular protein fractions obtained at various days p.i. from leaves producing *At*Phyt-EGFP (bold line). Intracellular protein fractions from *N. benthamiana* leaves infiltrated with *Agrobacterium* cells carrying the empty vector (pCambia1300, dashed line) and total protein fractions from leaves producing the inactive *At*Phyt-S553A-EGFP mutant (dotted line) served as controls. Fluorogenic peptide substrate Ac-YVAD-AFC was used at 30 μM to quantify the phytaspase activity. Relative rates of hydrolysis were determined as an increase of relative fluorescence units per hour (ΔRFU/h). Data represent the mean of three experiments ±SD. **(B)** Western blot analysis of *At*Phyt-EGFP in total extracts from *N. benthamiana* leaves producing the *At*Phyt-EGFP protein at various days p.i. The arrowhead points to position of full-length protein (~120 kDa). S553A, sample from leaves producing catalytically inactive *At*PhytS553A-EGFP protein at 3 d.p.i. Monoclonal anti-EGFP antibody was used for protein detection. Positions of molecular weight markers are indicated on the right. **(C)** Cleavage specificity of the proteolytic activity under study corresponds to that of *At*Phyt. Total protein extract obtained at day 4 p.i. was incubated with a panel of fluorogenic peptide substrates (30 μM). Ac-YVAD-AFC and Ac-IETD-AFC are the preferred *At*Phyt substrates (Chichkova et al., 2018). Relative rates of hydrolysis were determined as in **(A)**. Data represent the mean of three experiments ±SD. Specificity profiles with protein samples obtained at day 3 and 5 p.i. were similar to this one. **(D)** N-ethylmaleimide (2 mM), an inhibitor of VPE protease, does not interfere with hydrolysis of Ac-YVAD-AFC by total protein extracts obtained at day 4 p.i. from *At*Phyt-EGFP producing (*At*Phyt-EGFP) and non-producing (Empty vector) leaves. Relative rates of hydrolysis were determined as in **(A)**. Data represent the mean of two experiments ±SD. Similar results were obtained with a 3 d.p.i. sample. **(E,F)** The *At*Phyt-EGFP protein (wt, **E**) and its catalytically inactive mutant *At*PhytS553A-EGFP (S553A, **F**) are produced with similar efficiency in *N. benthamiana* leaves. Images were obtained at day 2 post-infiltration. Bar, 20 μm.

**Figure 7:**
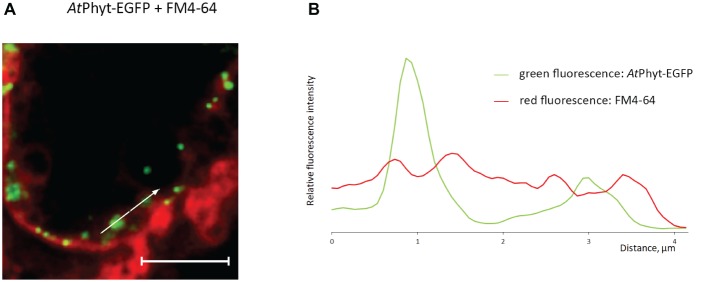
Vesicular membrane trafficking of *At*Phyt-EGFP in the course of internalization. **(A)** *At*Phyt-EGFP-producing *N. benthamiana* leaves were treated at day 3 p.i. with the FM4-64 fluorescent membrane-staining dye for 4 h and examined by confocal fluorescence microscopy. Bar, 5 μm. **(B)** Plotting fluorescence intensity for *At*Phyt-EGFP (green) and FM4-64 (red) along the line drawn through phytaspase-positive dots (shown by the arrow in **A**) demonstrates phytaspase encirclement with membranous structures.

The obtained plasmid constructs were introduced into *Agrobacterium tumefaciens* C58C1 or GV3101 cells. Transformed agrobacteria were infiltrated into *N. benthamiana* leaves using a blunt syringe or vacuum-infiltrated into seedlings (see below) in combinations described in legends (Figures 1–7). Agrobacteria carrying the empty vector (pCambia1300 or pLH7000) were used as a control, and were also added to the infiltration mix in the case of co-expression experiments to equalize plasmid ratio and bacterial load. At the indicated days post-infiltration (p.i.), leaves were examined by confocal fluorescence microscopy. Where indicated, treatment of leaves with antimycin A was performed by vacuum infiltration with water containing 10 μM antimycin A (Sigma, from 20 mM stock solution in ethanol). Control leaves were infiltrated with distilled water supplemented with an equivalent amount of ethanol. After an overnight incubation, confocal microscopy images were taken to determine fluorescence distribution in leaf tissue.

In parallel, leaves were subject to protein fractionation for subsequent determination of phytaspase activity. Apoplastic washes were obtained by low-speed (2,000 *g*) centrifugation of leaves at 4°C for 10 min. The leaf material was re-extracted with 20 mM MES buffer, pH 5.5, containing 100 mM NaCl and 25 μg/ml AEBSF, 2 μg/ml aprotinin, 5 μg/ml E-64 (all from Sigma), and 6 μg/ml leupeptin (MP Biomedicals) protease inhibitors, by vacuum infiltration and centrifugation, and the apoplastic washes were combined. After the separation, the residual leaf material was frozen in liquid nitrogen and disrupted in Minilys homogenizer (Bertin Instruments) using 1.6 mm ceramic beads by two 10 s bursts. An additional 10 s burst was performed after suspending the samples in B1 buffer (20 mM MES, 2 mM dithiothreitol, 0.1% Tween 20, 5% glycerol), pH 5.5, containing 50 mM NaCl and protease inhibitors (225 μl of the buffer per 25 mg of leaves). Debris was eliminated by 10 min centrifugation at 10,000 *g* at 4°C, and the supernatants (as well as the apoplastic washes) were taken for phytaspase activity determination. Leaves without prior separation of the apoplastic liquid were processed in an analogous fashion to obtain “total protein” samples. For western blot analysis and for glucose 6-phosphate dehydrogenase (G6PDH) activity determination, leaves were infiltrated with 20 mM MES buffer, pH 5.5, containing 100 mM NaCl and protease inhibitors. After separating the apoplastic protein fraction, the remaining leaf material (as well as the total/unwashed tissue) was ground in liquid nitrogen and extracted with 10 mM Tris-HCl buffer, pH 9.0, containing 0.2 M KCl, 30 mM MgCl_2_, 0.2 M sucrose, 10 mM 2-mercaptoethanol, and protease inhibitors. Western blot analysis of the EGFP- and mRFP-fused proteins was performed using monoclonal anti-EGFP 3A9 antibody (Sukhacheva et al., 2002) and polyclonal anti-mRFP antibodies (Abcam) as described (Chichkova et al., 2010).

For G6PDH activity determination (Yang et al., 2019), the intracellular protein and apoplastic fractions obtained from approximately 12 mg of leaf tissues were diluted 10- to 100-fold with 33 mM HEPES buffer, pH 7.5, containing 5 mM MgCl_2_, and 5 mM glucose 6-phosphate. As the protein fractions were obtained in different buffers, to equalize buffer conditions, the reaction mixtures were supplemented with the corresponding amounts of the complementary buffer prior to starting the reaction by the addition of NADP to a final concentration of 0.5 mM. G6PDH activity was determined spectrophotometrically, by measuring change in absorbance at 340 nm.

### Phytaspase Activity Quantification

Proteolytic activity of phytaspases in apoplastic washes, intracellular protein fractions, and in total *N. benthamiana* leaf extracts was determined using either the Ac-VEID-AFC [AFC, 7-amino-4-(trifluoromethyl) coumarin] fluorogenic peptide substrate (for *Nt*Phyt and for endogenous *N. benthamiana* phytaspase), or for *At*Phyt, the Ac-YVAD-AFC substrate (both from California Peptide). Fluorogenic peptide substrates Ac-VAD-AFC, Ac-VDVAD-AFC, Ac-LEHD-AFC, Ac-WEHD-AFC, Ac-DEVD-AFC (all from Calbiochem), Ac-VNLD-AFC (California Peptide), Ac-STATD-AFC (Bachem), and Ac-IETD-AFC (Anaspec) were used to assess cleavage specificity. Peptide substrates were used at a final concentration of 30 μM. Protein samples were 10- to 15-fold diluted before activity measurements. Kinetic measurements of relative fluorescence increase were performed in B1 buffer, pH 5.5 (for tobacco phytaspases) or pH 6.5 (for *At*Phyt), containing 0.5 M NaCl and protease inhibitors at 28°C. Where indicated, N-ethylmaleimide (Sigma) was added to the reaction mixtures to a final concentration of 2 mM. The Fluoroskan Ascent reader (Thermo Fisher Scientific) equipped with 390 nm excitation and 510 emission filters was used to quantitate fluorescence intensities. Data are presented as means ± SD from three independent experiments. Statistical significance was analyzed using Student’s *t*-test. *p* < 0.01 were considered significant.

### Treatment of *N. benthamiana* Seedlings

Seven-day-old sterile *N. benthamiana* seedlings grown in solid growth medium (2.17 g/L Murashige and Skoog basal salt mixture, 0.5 g/L MES, 10 g/L glucose, 0.8% agar, pH 5.7) were vacuum infiltrated with transformed agrobacteria. Three days after infiltration, seedlings were detached and submerged in water containing 10 μM antimycin A for 3 h. Control seedlings were submerged in water supplemented with an equivalent amount of ethanol. After that, seedlings were stained with Evans Blue (Sigma) according to Minina et al., 2013. Briefly, detached seedlings were vacuum infiltrated with fresh aqueous solution of 0.5% Evans Blue and incubated for 15 min at room temperature, then washed three times for 15 min with water, slightly shaking to remove unbound dye. Chlorophyll was removed by incubating samples in 96% ethanol for 10 min at room temperature twice, then ethanol was removed by washing with 70, 50, 20% ethanol and water, consequently.

### Confocal Fluorescence Microscopy

Samples were studied using Nikon C2+ confocal microscope based on Nikon Eclipse Ti (Nikon, Japan) inverted body equipped with 60× NA 1.2 plan-apochromat water immersion lens with the working distance of 300 μm and 402, 488, and 562 nm lasers used for excitation of DAPI, EGFP, and mRFP fluorescence, respectively. Fluorescence was detected using 560 nm dichroic mirror and 525/50 and 595/40 nm blocking filters for EGFP and mRFP-tagged proteins, respectively. FM4-64 fluorescence was also recorded using 595/40 nm filter. The same pinhole diameter setting of 60 μm was used for both channels, resulting in optical sectioning of 650 nm for green and red channels. Deconvolution was performed using Richardson-Lucy algorithm implemented in the microscope controlling software Nis Elements AR (Nikon, Japan). FM4-64 (Molecular Probes) was infiltrated into leaves at a 5 μM concentration in water. Images were taken 4 h p.i. Data were reproducible over at least three independent experiments.

## Results

### Stress-Induced Internalization of *N. tabacum* Phytaspase Depends on Clathrin-Mediated Endocytosis and is Specific

*N. tabacum* phytaspase (*Nt*Phyt) is known to accumulate in the apoplast of healthy leaves and to re-localize toward the cell interior in response to PCD-inducing triggers (Chichkova et al., 2010). To follow *Nt*Phyt trafficking, the *Nt*Phyt-EGFP protein was transiently produced in *N. benthamiana* leaves by agroinfiltration. Fluorescence microscopy examination of the infiltrated leaves confirmed the production of the target protein, with no fluorescence occurring in the cell interior (Figure 1A). Triggering oxidative stress-induced cell death in these leaves by treatment with 10 μM antimycin A resulted in the re-distribution of *Nt*Phyt-EGFP, visible as the formation of multiple small dots within the cell (Figure 1B). Western blot analysis of protein samples from control (mock-treated) and antimycin A-treated leaves did not reveal degradation of the *Nt*Phyt-EGFP protein in response to antimycin A treatment (Figure 1C). To verify the initial apoplastic localization of *Nt*Phyt, a plasma membrane protein marker EGFP-LTI6b (Cutler et al., 2000) was transiently produced together with *Nt*Phyt-mRFP in *N. benthamiana* leaves (Figure 1D). In plasmolysed leaf samples, the *Nt*Phyt-mRFP red fluorescence was visualized in the space between two plasma membranes (green) of the adjacent epidermal cells (Figures 1E–G). This observation was also supported by measuring fluorescence intensities across the boundaries of the two adjacent plant cells (Figure 1H). The *Nt*Phyt-mRFP protein responded to treatment with antimycin A similarly to the *Nt*Phyt-EGFP fusion, i.e., by forming small dots within the cell (Figures 1I,J).

To verify the involvement of clathrin-mediated endocytosis in the observed antimycin A-induced re-localization of *Nt*Phyt, mRFP-Hub1 was produced together with *Nt*Phyt-EGFP in *N. benthamiana* leaves. Hub1 represents the C-terminal fragment of *A. thaliana* clathrin heavy chain 1, which acts, upon over-production in *A. thaliana* and *N. benthamiana*, in a dominant negative fashion to specifically inhibit clathrin-mediated endocytosis (Liu et al., 1995; Dhonukshe et al., 2007; Kitakura et al., 2011; Li and Pan, 2017). In the presence of mRFP-Hub1, no stress-induced re-localization of *Nt*Phyt-EGFP was observed (Figures 1K–P). Thus, similar to results from previous studies (Chichkova et al., 2010; Trusova et al., 2019), oxidative stress-induced PCD caused retrograde transport of *Nt*Phyt-EGFP from the apoplast to the cell interior, which could be efficiently blocked by inhibiting clathrin-mediated endocytosis.

One could imagine that internalization of *Nt*Phyt by means of clathrin-mediated endocytosis could be achieved either through specific recognition of the cargo or non-specific capturing of the apoplastic fluid by the newly forming clathrin-coated pits. Availability of an inert soluble apoplastic protein marker would be helpful to distinguish between these possibilities. To construct such a marker, we fused the *Nt*Phyt signal peptide (SP, amino acid residues 1–24) to the N-terminus of the mRFP protein to drive secretion of the fluorescent protein into the apoplast and produced the resultant SP-mRFP protein in *N. benthamiana* leaves by agroinfiltration. Figure 2A shows that mRFP fluorescence was detectable at the cell borders in the infiltrated leaves. After separating the proteins into apoplastic and intracellular fractions, mRFP was visualized as an apoplastic protein by western blot analysis (Figure 2B). This was in sharp contrast to the behavior of free mRFP (without signal peptide) produced in *N. benthamiana* leaves. While mRFP fluorescence was located to the cell periphery and the nucleus (Figure 2C), separating the proteins into apoplastic and intracellular fractions expectedly revealed free mRFP as an intracellular protein by western blot analysis (Figure 2D). Of note also, the results of western blot analyses (Figures 2B,D) demonstrate the absence of appreciable cross-contamination between apoplastic and intracellular fractions. To further confirm this notion, enzymatic activity of glucose 6-phosphate dehydrogenase (G6PDH), an intracellular protein (Debnam and Emes, 1999), was quantified in both fractions. Figure 2E shows that the majority of the G6PDH activity was present in the intracellular fraction, with less than 5% of the total activity observed in the apoplastic fraction.

As production of SP-mRFP was found to generate a reliable and soluble apoplastic protein marker, we aimed to use it to assess specificity of phytaspase internalization. Co-production of *Nt*Phyt-EGFP and SP-mRFP in *N. benthamiana* leaves resulted in co-localization of both proteins in the apoplast under non-stressed conditions (Figures 2F,H,J). Antimycin A-induced cell death caused the expected shift of the *Nt*Phyt-EGFP fluorescence toward the cell interior (Figure 2G). In contrast, mRFP fluorescence retained its extracellular localization (Figures 2I,K).

We concluded that clathrin-mediated endocytosis of phytaspase proceeds through a specific recognition step, most likely involving a phytaspase receptor at the plasma membrane of plant cells, rather than by a non-specific fluid-phase uptake.

### Internalized Phytaspase Retains Proteolytic Activity

To determine if phytaspase retains its proteolytic activity upon PCD-induced internalization, we analyzed the behavior of endogenous *N. benthamiana* phytaspase (*Nb*Phyt) first. Apoplastic washes were obtained from *N. benthamiana* leaves containing endogenous phytaspase only (in the absence of *Nt*Phyt-EGFP production) and from leaves producing mRFP-Hub1, either treated with antimycin A or untreated. Phytaspase activity was quantified in these apoplastic washes (“apoplast”), as well as in residual leaf tissue (“intracellular fraction”) and in the total (unwashed) tissues (“total”). The preferred fluorogenic peptide substrate of *Nt*Phyt, Ac-VEID-AFC (Chichkova et al., 2010), was used for proteolytic activity measurements. In the “endogenous *Nb*Phyt only” leaves in the absence of stress, the majority of phytaspase activity was detected in the apoplast. Antimycin A-induced oxidative stress resulted in a dramatic re-localization of the phytaspase proteolytic activity to inside the cell (Figure 3A, upper panel). Production of mRFP-Hub1 did not have an appreciable effect on the level and distribution of phytaspase activity in healthy *N. benthamiana* tissues. However, upon the induction of oxidative stress, the inhibition of clathrin-mediated endocytosis precluded the accumulation of phytaspase activity inside the cells (Figure 3A, lower panel).

Analogous fractionation and proteolytic activity measurements were then performed for *N. benthamiana* leaves transiently producing *Nt*Phyt-EGFP, leaves producing *Nt*Phyt-EGFP together with mRFP-Hub1, either treated with antimycin A or not. The distribution of proteolytic activity of the ectopically produced *Nt*Phyt-EGFP was found to be similar to that of endogenous enzyme in regards to the stress-induced re-localization to inside the cells (Figure 3B, upper panel) and the dependence of this retrograde trafficking on clathrin-mediated endocytosis (Figure 3B, lower panel). The only variance was the apparently less pronounced difference between the proteolytic activity levels in the apoplast versus the intracellular protein fractions observed upon the *Nt*Phyt-EGFP overproduction.

An increase in the phytaspase-specific proteolytic activity observed upon *Nt*Phyt-EGFP overproduction suggests that this activity belongs to *Nt*Phyt-EGFP. To further verify this assumption, total extracts were prepared from leaves infiltrated with agrobacteria carrying either the wild type *Nt*Phyt-EGFP – encoding plasmid, or the *Nt*Phyt-S537A-EGFP – encoding plasmid driving production of catalytically inactive phytaspase mutant (Chichkova et al., 2010), or the empty vector. Determination of the Ac-VEID-AFC – hydrolyzing activities in total extracts demonstrated a marked increase of the proteolytic activity in the case of wild type *Nt*Phyt-EGFP production, relative to the empty vector control (Figure 3C). Production of the inactive *Nt*Phyt-S537A-EGFP protein, on the other hand, failed to increase proteolytic activity over the background level, although both proteins were overproduced to a similar level judging by western blot analysis of the extracts (Figure 3D) and fluorescence intensities of infiltrated leaves (Figures 3E,F).

Based on the results obtained both with the endogenous and ectopically produced phytaspases, we conclude that induction of cell death does not markedly change the overall level of phytaspase activity in plant tissues. Rather, it causes the re-distribution of proteolytically active phytaspase from the apoplast to inside the cells through the retrograde trafficking step that is critically dependent on clathrin-mediated endocytosis.

### Correlating *Nt*Phyt Activity and Trafficking with Plant Cell Death

Previously, by using transgenic tobacco plants with up- and down-regulated phytaspase activity, *Nt*Phyt has been demonstrated to promote plant cell death induced by biotic and abiotic stresses, with concomitant retrograde transport of the enzyme (Chichkova et al., 2010). We, therefore, strived to evaluate whether transient *Nt*Phyt overproduction and/or inhibition of *Nt*Phyt internalization could affect plant cell viability. To address these issues, seven-days-old *N. benthamiana* seedlings were vacuum-infiltrated with agrobacteria carrying either empty vector, or the *Nt*Phyt-EGFP – encoding plasmid, or the mRFP-Hub1 – encoding plasmid, or a combination thereof, or the *Nt*Phyt-S537A-EGFP – encoding plasmid. Cell death in the cotyledons was examined by staining with Evans Blue both with and without treatment of seedlings with antimycin A. As shown in Figure 4, antimycin A-induced oxidative stress caused extensive cell death (A,B), as expected. On the other hand, transient production of *Nt*Phyt-EGFP predisposed cells to death even in the absence of antimycin A treatment (Figure 4, compare images A and E), whereas the proteolytically inactive phytaspase mutant failed to do so (Figure 4, compare images E and I). The pro-death effect of *Nt*Phyt-EGFP production was further enhanced by the subsequently applied oxidative stress (Figure 4F). Notably in the presence of mRFP-Hub1, both the antimycin A-induced and *Nt*Phyt-EGFP-promoted cell death was attenuated (Figures 4D,G,H). These results are in line with pro-death proteolytic activity of *Nt*Phyt, and furthermore they highlight the importance of clathrin-mediated endocytosis for accomplishment of stress-induced and *Nt*Phyt-promoted death of plant cells.

### Clathrin-Mediated Internalization of *A. thaliana* Phytaspase in a Model System

The *A. thaliana* phytaspase (*At*Phyt) is distinct from the characterized phytaspases in other plant species in a number of ways (Chichkova et al., 2018). In particular, Trusova et al., 2019 recently documented the unexpected mobility of *At*Phyt-EGFP produced in *N. benthamiana* leaves. The recombinant protein expressed in *N. benthamiana* epidermal cells by agroinfiltration initially localizes along cell borders. However after 2 days post infiltration (p.i.), *At*Phyt-EGFP was spontaneously (in the absence of any additional treatment) re-imported into *N. benthamiana* cells (Figures 5A,B). This differs significantly from the behavior of the same protein in *A. thaliana* epidermal cells and of *Nt*Phyt-EGFP protein in the non-stressed *N. benthamiana* cells. Notably, EGFP fluorescence in tissues rapidly declined on days 4 and 5 p.i. (Figures 5C,D). Spontaneous *At*Phyt-EGFP internalization in this system occurred in the absence of death symptoms in leaf tissue (Trusova et al., 2019) and was critically dependent on clathrin-mediated endocytosis (Figures 5E–P). When clathrin-mediated endocytosis was suppressed by co-production of the mRFP-Hub1 inhibitor, no internalization of the enzyme occurred at days 3 and 4 (Figures 5F,G), and apoplastically “arrested” *At*Phyt-EGFP persisted outside the cells at day 5 p.i. (Figures 5E–H).

Co-production of the SP-mRFP protein together with *At*Phyt-EGFP in *N. benthamiana* leaves revealed that the secreted mRFP retained its apoplastic localization when the internalization of *At*Phyt-EGFP was essentially complete (Figures 5Q–T). This indicates that the spontaneous internalization of *At*Phyt-EGFP occurs specifically, similar to the stress-induced internalization of *Nt*Phyt-EGFP described above.

To determine whether the internalized *At*Phyt-EGFP protein retains its proteolytic activity, intracellular proteins were obtained from the *At*Phyt-EGFP-producing leaves on different days following infiltration. *At*Phyt proteolytic activity was quantified using the preferred *At*Phyt fluorogenic peptide substrate Ac-YVAD-AFC (Chichkova et al., 2018), which is also a sub-optimal substrate for the tobacco phytaspase. Internalization of *At*Phyt-EGFP occurring at days 3 and 4 p.i. was followed by an increase of *At*Phyt activity inside the cells (Figure 6A). At day 5 p.i. this intracellular proteolytic activity was still clearly detectable, although somewhat decreased.

Western blot analysis with an anti-EGFP antibody of the total protein extracts obtained from the *At*Phyt-EGFP-producing leaves on different days following infiltration revealed the accumulation of the *At*Phyt-EGFP protein at days 2 and 3 p.i., which was followed by a sharp decline of the protein level at days 4 and 5 p.i. (Figure 6B). Concomitantly with the *At*Phyt-EGFP disappearance, a protein of approximately 28 kDa was accumulated during days 3–5 p.i. The absence of EGFP fluorescence in leaf tissues at day 5 p.i. (Figure 5D) may indicate that this newly formed product corresponds to a non-fluorescent EGFP fragment. Meanwhile, the presence of the YVADase activity in the 5 day p.i. sample suggests that either active *At*Phyt survives after the EGFP detachment, or the observed proteolytic activity does not belong to *At*Phyt.

To clarify this issue, a panel of 10 synthetic fluorogenic peptide substrates used previously to characterize *At*Phyt cleavage specificity (Chichkova et al., 2018) was employed. In full agreement with the established *At*Phyt specificity, protein samples obtained at days 3, 4, and 5 following infiltration hydrolyzed Ac-YVAD-AFC and Ac-IETD-AFC substrates most efficiently (Figure 6C), whereas low level of cleavage or no hydrolysis at all was observed with other fluorogenic peptides.

As an alternative, the YVADase activity in the intracellular protein extracts could belong to vacuolar processing enzyme (VPE), a cysteine-dependent proteinase known to recognize YVAD-based substrates and inhibitors (Hatsugai et al., 2004; Rojo et al., 2004). However, hydrolysis of the Ac-YVAD-AFC substrate by protein extracts was not affected by the presence of 2 mM N-ethylmaleimide, a VPE inhibitor (Hiraiwa et al., 1999) (Figure 6D). Finally, when the catalytically inactive *At*PhytS553A-EGFP mutant was transiently produced in *N. benthamiana* leaves (see Figure 6B for the relative level of production), no proteolytic activity above the background level was observed with the Ac-YVAD-AFC substrate using total leaf extracts obtained at any time point (Figure 6A). Fluorescence microscopy examination of agroinfiltrated tissues confirmed efficient production of both proteins as well (Figures 6E,F).

Taken together, these results suggest that the enhanced intracellular YVAD-AFC-hydrolyzing proteolytic activity belongs to the transiently produced *At*Phyt, and that the activity survives within the cell for several days.

To further examine the retrograde vesicular membrane trafficking of phytaspase at the healthy cell background, *At*Phyt-EGFP-producing *N. benthamiana* leaves at 3 days p.i. (when the *At*Phyt-EGFP protein was largely intact) were treated with FM4-64 fluorescent membrane-staining dye. Prolonged incubation with FM4-64 allowed the dye to become endocytosed and stain intracellular membranous compartments (Jelínková et al., 2010), whereas *At*Phyt-EGFP was visualized as intracellular green dots (Figure 7A). Measuring green and red fluorescence intensities along the line drawn through phytaspase-positive dots (direction is shown by the arrow in Figure 7A) revealed that the internalized *At*Phyt-EGFP (green) is entrapped within the membranous (FM4-64-positive, red) structures (Figure 7B). This is consistent with the vesicular membrane mechanism of delivery of active phytaspase into plant cells.

## Discussion

Clathrin-mediated endocytosis was recently shown to drive PCD-induced retrograde transport of phytaspases, plant cell death-related proteases, from the apoplast into the plant cells (Trusova et al., 2019), raising new important questions regarding mechanisms and functional consequences of this unanticipated trafficking pathway. What happens to the proteolytic activity of the enzyme upon internalization? Is the uptake of phytaspases specific, or any soluble apoplastic protein will become internalized upon induction of cell death? Will interference with the phytaspase uptake compromise cell death? Here, we addressed these questions using *N. benthamiana* leaves either containing endogenous phytaspase only, or overproducing *Nt*Phyt tagged with EGFP or mRFP that allowed to follow phytaspase re-distribution with the aid of fluorescence microscopy in parallel with the determination of a peculiar Asp-specific activity of phytaspases. *N. benthamiana* leaves either treated with antimycin A to trigger oxidative stress-induced PCD and phytaspase internalization, or mock-treated were used in these studies.

By separating proteins into apoplastic and intracellular fractions and by quantifying phytaspase proteolytic activity using fluorogenic peptide substrates of phytaspases, we showed that retrograde transport of phytaspases is accompanied by the shift of phytaspase activity toward the cell interior. When internalization of phytaspases was blocked by co-production of Hub, the inhibitor of clathrin-mediated endocytosis, phytaspase proteolytic activity was retained within the apoplast. It is important to note that the overall level of phytaspase activity in leaf tissues was not diminished after phytaspase internalization. Efficient degradation within the vacuole is a frequent outcome for endocytosed proteins (Kleine-Vehn et al., 2008; Korbei and Luschnig, 2013; Claus et al., 2018; Reynolds et al., 2018). However, the internalized phytaspases are evidently able to deviate from the degradation pathway.

Therefore, our results indicate that clathrin-mediated endocytosis accomplishes the delivery of active phytaspases inside the plant cell. The process of phytaspase internalization appears to be specific, as the apoplastic mRFP protein (originating from the signal peptide-mRFP precursor) retained its extracellular localization both before and after the induction of cell death. This suggests that a receptor for phytaspases exists in the plant cell plasma membrane. Indeed, clathrin-mediated endocytosis targets proteins that localize at the plasma membrane and possess cytoplasmic domains, to which clathrin is recruited with the aid of adapter proteins (Traub, 2009; Jackson et al., 2010; Chen et al., 2011; Traub and Bonifacino, 2013; Paez Valencia et al., 2016). Phytaspases, however, are soluble proteins that can be easily obtained in apoplastic wash. Therefore, an interface between phytaspases and clathrin endocytic machinery appears to be necessary. Identification of such a receptor would be an interesting task for the future.

The known involvement of phytaspases in the accomplishment of stress-induced plant cell death (Chichkova et al., 2004, 2010; Reichardt et al., 2018) has pushed us to explore whether prevention of phytaspase internalization would promote cell viability under the unfavorable conditions. We found that interruption of phytaspase uptake through inhibition of clathrin-mediated endocytosis correlated with alleviation of cell death induced by oxidative stress (antimycin A treatment) and by *Nt*Phyt overproduction. These findings appear to provide the first indication of the importance of clathrin-mediated endocytosis for stress-induced death of plant cells. Also, inability of the catalytically inactive *Nt*Phyt mutant, in contrast to the wild type enzyme, to cause cell damage further emphasizes the importance of the *Nt*Phyt proteolytic activity for promoting plant cell death. However, although the obtained data are in line with the current model of phytaspase participation in plant PCD (Vartapetian et al., 2011; Chichkova et al., 2012; Trusova et al., 2019), further efforts are obviously required for detailed characterization of the underlying mechanisms.

The second experimental system used in this study, the “*At*Phyt-EGFP in *N. benthamiana*” model, can of course be regarded as artificial and results obtained with this system should be interpreted with some caution. Indeed, we do not yet understand why internalization of the *At*Phyt-EGFP protein begins spontaneously in *N. benthamiana* epidermal cells after a 2-day lag period. A requirement for high level pre-accumulation of the *At*Phyt-EGFP protein within the apoplast to drive internalization could possibly account for the observed phenomenon. Also, what is the difference between *At*Phyt-EGFP and *Nt*Phyt-EGFP that allows the former to enter cells spontaneously, while the latter requires a PCD-inducing stimulus for internalization?

Despite these open questions, the behaviors of *Nt*Phyt and *At*Phyt in *N. benthamiana* leaves share a number of features, as shown by the employment of clathrin-mediated endocytosis for their specific internalization. As spontaneous uptake of *At*Phyt-EGFP does not appear to be associated with *N. benthamiana* cell death (Trusova et al., 2019), the *At*Phyt-EGFP model may offer an opportunity to address details of phytaspase internalization in live plant cells, in the absence of major complicating perturbations occurring in dying cells. With this approach, co-localization of the internalized *At*Phyt-EGFP protein with intracellular membranous vesicles was observed, which is consistent with the endocytic entry pathway for phytaspases.

The described localization dynamics of phytaspases is atypical for plant subtilisin-like proteases, for which secretion into the apoplast was considered to be the end point of their trafficking. In this regard, it would be interesting to learn whether phytaspases represent a rare exception in their retrograde vesicular trafficking, or perhaps other plant subtilases behave in a similar manner under certain conditions.

## Data Availability

The raw data supporting the conclusions of this manuscript will be made available by the authors, without undue reservation, to any qualified researcher.

## Author Contributions

AV, NC, and ST designed the study and directed the research. ST, AT, SG, EM, RG, and NC performed experiments. NC, ST, AT, SG, EM, RG, and AV analyzed and interpreted the data. AV wrote the manuscript with contribution from NC. All the authors read and approved the manuscript.

### Conflict of Interest Statement

The authors declare that the research was conducted in the absence of any commercial or financial relationships that could be construed as a potential conflict of interest.
